# Identification of lipid A deacylase as a novel, highly conserved and protective antigen against enterohemorrhagic *Escherichia coli*

**DOI:** 10.1038/s41598-019-53197-z

**Published:** 2019-11-19

**Authors:** Maricarmen Rojas-Lopez, Manuele Martinelli, Valentina Brandi, Grégory Jubelin, Fabio Polticelli, Marco Soriani, Mariagrazia Pizza, Mickaël Desvaux, Roberto Rosini

**Affiliations:** 1 GSK, Via Fiorentina 1, 53100 Siena, Italy; 2 Université Clermont Auvergne, INRA, UMR454 MEDiS, F-63000 Clermont-Ferrand, France; 3 Roma Tre University, Department of Sciences, Viale G. Marconi 446, 00146 Rome, Italy; 4 National Institute of Nuclear Physics, Roma Tre Section, Via della Vasca Navale 84, 00146 Rome, Italy; 5 Present Address: Department of Medicine, Division of Infectious Diseases, Massachusetts General Hospital; Department of Microbiology and Immunobiology, Harvard Medical School, Boston, Massachusetts USA; 6 Present Address: ReiThera Srl, Via di Castel Romano 100, 00128 Roma, Italy

**Keywords:** Immunology, Vaccines

## Abstract

Enterohemorrhagic *E. coli* (EHEC) is a major cause of large outbreaks worldwide associated with hemorrhagic colitis and hemolytic uremic syndrome. While vaccine development is warranted, a licensed vaccine, specific for human use, against EHEC is not yet available. In this study, the reverse vaccinology approach combined with genomic, transcriptional and molecular epidemiology data was applied on the EHEC O157:H7 genome to select new potential vaccine candidates. Twenty-four potential protein antigens were identified and one of them (MC001) was successfully expressed onto Generalized Modules for Membrane Antigens (GMMA) delivery system. GMMA expressing this vaccine candidate was immunogenic, raising a specific antibody response. Immunization with the MC001 candidate was able to reduce the bacterial load of EHEC O157:H7 strain in feces, colon and caecum tissues after murine infection. MC001 is homologue to lipid A deacylase enzyme (LpxR), and to our knowledge, this is the first study describing it as a potential vaccine candidate. Gene distribution and sequence variability analysis showed that MC001 is present and conserved in EHEC and in enteropathogenic *E. coli* (EPEC) strains. Given the high genetic variability among and within *E. coli* pathotypes, the identification of such conserved antigen suggests that its inclusion in a vaccine might represent a solution against major intestinal pathogenic strains.

## Introduction

Enterohemorrhagic *Escherichia coli* (EHEC) is an anthropozoonotic and etiological agent of diarrheal disease and hemorrhagic colitis. EHEC infections occur mainly in developed countries and the strains most often implicated in outbreaks are the O157:H7 and the big six non-157 serotypes (O26:H11, O45:H2, O103:H2, O111:H8, O121:H19 and O145:H28)^1–3^. Ruminants are the main reservoir of EHEC and therefore the infection mainly occurs from fecal contamination of food products^4^. EHEC strains are characterized by the expression of the Shiga toxin (Stx), the hallmark of the pathotype. Furthermore, some strains also carry the enterocyte effacement (LEE) locus that encodes the Type III secretion system (T3SS) responsible for the generation of attachment and effacing (A/E) lesion on the intestinal microvilli^1^.

The complications arising from EHEC include hemorrhagic colitis, the development of the hemolytic uremic syndrome (HUS) and renal failure^5^. Although the use of antibiotics remains the gold standard for the treatment of bacterial diseases, they are not recommended to treat EHEC infections^4,6^. Antibiotic treatment could lead to cellular damages by increasing the production of Stx, causing its release into the blood stream and further worsening the disease outcome^7^.

In general, the increasing burden of these *E. coli* diarrheal diseases, the emergence of hybrids strains, and the increasing annual cost for the health care systems reflect the need to develop effective therapeutic and preventive strategies. Among these, vaccination is the most promising strategy to control disease not only for EHEC but also for others pathogenic *E. coli* strains^2,3,8,9^.

So far, several vaccine candidates have been identified by different approaches. Virulence factors expressed as recombinant proteins such as Stx, intimin, *E. coli* secreted protein A (EspA), and avirulent ghost cells of EHEC O157:H7 have been tested using different immunization routes and adjuvant combinations in several animal models with encouraging results^10^. A recent *in silico* approach aimed to develop DNA based vaccine identified new EHEC antigens, including among others a putative pilin subunit, T3SS structural protein (*escC*) gene, and an outer membrane protein encoded by the bacteriophage Bp933W gene *lomW*^11,12^. Additionally, previous studies have identified new promising vaccine candidates demonstrating the potential of exploiting the reverse vaccinology concept^11–16^. So far, this strategy has been performed on a completely sequence genome of an extraintestinal neonatal meningitis *E. coli* isolate (NMEC) leading to the identification of 230 potential antigens. Among these, a conserved zinc metallopeptidase, SslE, was one of the most protective antigens by conferring protection in three different murine models^15,17,18^.

In addition to the available technologies, new vaccine development strategies have been recently explored. These innovations ideally serve to make vaccine production simpler, more cost effective, and improve antigen presentation and immune response^19^. Outer membrane vesicles are one of these systems employed for vaccine development against Gram-negative bacteria. These microorganisms release native outer membrane vesicles (NOMV) that are rich in outer membrane lipids, outer membrane and periplasmic proteins, and are subsequently presented to the immune system in their natural conformation^20^. NOMV-based vaccines have been largely employed against the organism from which they are recovered^21–23^ or to express and deliver heterologous antigens^24–26^. However, in native conditions NOMV are recovered in small quantities but *E. coli* strains can be genetically modified by deletion of the *tolR* gene to enhance the level of vesicle production^27^. This system has been successfully used for expressing properly folded membrane-associated recombinant antigens and to induce functional immune responses^24^. Recently, this antigen delivery approach, also known as GMMA (Generalized Modules for Membrane Antigens), has been successfully implemented for vaccine development^28–30^.

The main goal of this work was to identify novel antigens as potential vaccine candidates against infections caused by EHEC, using GMMA as delivery system. Our study led to the identification of a new potential vaccine candidate present in EHEC O157:H7 strains able to reduce intestinal bacterial colonization in mice.

## Results

### Identification of vaccine candidates by reverse vaccinology

**Figure 1:**
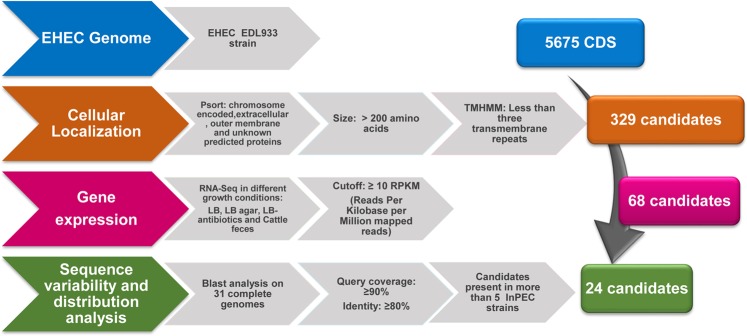
Reverse vaccinology strategy applied for vaccine candidate selection in EHEC O157:H7. Schematic representation of the *in-silico* pipeline used for vaccine candidate selection. The 5675 coding DNA sequences (CDS) encoded on the chromosome of EHEC O157:H7 EDL933 strain were analyzed to identify potential antigens based on their subcellular localization (PSORT). The selection included proteins greater than 200 amino acids and with fewer than 3 transmembrane repeats determined by the TMHMM algorithm. RNA-Seq data were used to find expressed candidate genes. Gene variability and distribution analysis of 68 potential antigens on 31 complete *E. coli* genomes was performed to select those present (query coverage: ≥ 90%) and conserved (sequence identity ≥ 80%) in more than 5 different intestinal pathogenic *E. coli* strains.

To identify potential antigens in the EHEC O157:H7 EDL933 prototype strain, the reverse vaccinology approach was applied by combining genomic analysis with transcriptional and molecular epidemiology data as summarized in Fig. 1. The PSORT algorithm was applied to predict the subcellular localization of the 5675 coding sequences (CDS) annotated in the genome. Chromosomal genes encoding for proteins predicted to be secreted, surface-exposed, outer membrane-associated or with an unknown subcellular localization, were selected. In addition, only genes encoding for proteins of at least 200 amino acids in length and with fewer than 3 transmembrane domains (as determined by the TMHMM algorithm) were included. This analysis resulted in 329 potential vaccine candidates (Table S1) and the transcription level of these genes was analyzed using the RNA-Seq database available in the NCBI Sequence Read Archive (SRA). The RNA-Seq dataset was generated using EHEC EDL933 strain grown in LB, LB with antibiotics, LB-agar media and cattle feces^31^. Sixty-eight of the 329 identified CDSs showed an absolute index number of ≥10 RPKM (reads per kilobase per million mapped reads) in at least one of the four growth conditions analyzed (Table S2). The 68 genes were then analyzed for gene distribution and variability in a panel of 31 complete genomes. Twenty-four genes were present and conserved in more than 5 intestinal pathogenic *E. coli* strains (query coverage: ≥90%; sequence identity ≥80%) (Fig. 1 and Fig. S1). The 24 genes encoding for potential vaccine antigens were cloned in *E. coli*, expressed and purified as recombinant His-tagged fusion proteins. Twelve were successfully purified in their soluble form, while 12 were insoluble (Table S3). The 24 recombinant proteins were then used to immunize mice to generate antigen-specific polyclonal antibodies. These antibodies were subsequently tested in Western Blot analyses to assess the expression level of the corresponding potential candidates in the EHEC O157:H7 strain whole cell extract, leading to the identification of 17 expressed proteins in standard laboratory growth conditions, of which five exhibited high protein expression (Table S3). These five candidates included four colonization/virulence factors and one putative outer membrane protein. Among these expressed proteins the putative outer membrane candidate (MC001) showed also a higher RNA expression level in two different growth conditions (Table S3). Considering the higher level of expression and all the aforementioned criteria, we selected MC001 for further characterization.

### Antigen delivery in Generalized Modules for Membrane Antigens (GMMA)

**Figure 2:**
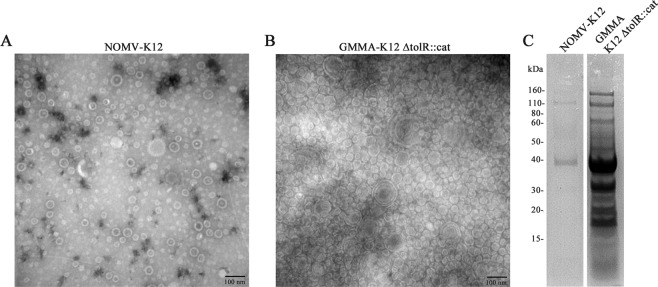
*E. coli* K12 engineering to generate Generalized Modules for Membrane Antigens (GMMA). NOMV and GMMA were isolated by ultracentrifugation from supernatants of *E. coli* K12 WT and K12 ∆*tolR::cat* (**A**) Negative staining of native NOMV released from a wild-type *E. coli* K12 observed by transmission electron microscopy (TEM). NOMV from K12 WT strain appeared as closed spherical particles and homogeneous in shape with a size ranging from 20 to 100 nm. (**B**) Negative staining of GMMA produced by the K12 *tolR::cat* (K12*tolR::cat*) strain analyzed by TEM GMMA from *tolR* mutant (size ranging from 20 to 200 nm) (magnification 120,000x). (**C**) SDS-PAGE (4–12% bis-tris polyacrylamide) of membrane vesicles (NOMV and GMMA) purified from 75 ml of culture supernatants. Total protein content was quantified and 50 μg of GMMA obtained from K12 ∆*tolR::cat* sample was loaded into the SDS-PAGE gel. An equivalent volumetric amount of NOMV from K12 WT obtained from 75 mL of supernatant was loaded. The *tolR* mutant showed an extensive protein profile in the supernatant compared to wild type.

**Figure 3:**
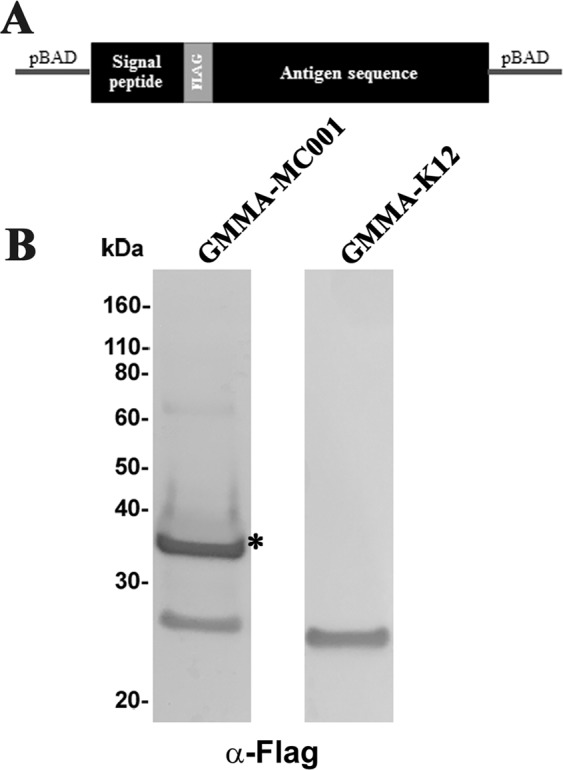
Antigen delivery into GMMA. (**A**) Schematic representation of the candidate cloning strategy. The coding sequence of the MC001 potential antigen was cloned into a pBAD vector incorporating its native signal peptide. A FLAG-tag was inserted after the signal peptide of the construct. (**B**) Western blot of GMMA preparation expressing the MC001 candidate purified from the K12 *tolR::cat* mutant, using an anti-FLAG antibody. Asterisks (*) indicate the expected molecular size of MC001.

When overexpressed in *E. coli* the MC001 protein was localized into the outer membrane and was purified from the recombinant strain only in its insoluble form. To keep the protein in its natural conformation, we investigated the possibility to overexpress it in *E. coli* K12 outer membrane vesicles. First, to identify the *E. coli* strain in which the candidate could be better expressed, native vesicles (NOMV) released by the *E. coli* K12 wild-type strain and by a *E. coli* K12 strains in which the *tolR* gene has been deleted (K12 ∆*tolR*::*cat*) to induce an overblebbing phenotype (GMMA)^24^, were purified and characterized further. The comparison of the total protein content from vesicles derived from the two strains showed that yield of the *tolR* mutant strain was 25-fold higher compared to the wild-type strain (Fig. 2C). By transmission electronic microscopy (TEM), the NOMV from the wild-type strain appeared as closed spherical particles ranging from 20 to 100 nm in diameter (Fig. 2A), while the GMMA from *tolR* mutant ranged from 20 to 200 nm (Fig. 2B). Based on these data, we selected the GMMA as system for the overexpression of the MC001 vaccine candidate. MC001 was cloned in frame onto pBAD plasmid and a FLAG-tag inserted after signal peptide sequence (Fig. 3A). The generated construct (pBAD-MC001F) was transformed into the *tolR* mutant and the expression and localization of the vaccine candidate into GMMA were evaluated by western blot on the vesicle preparations using the anti-FLAG antibody for detection. As shown in Fig. 3B, MC001 was successfully expressed and localized in the GMMA.

### Immunization with GMMA overexpressing MC001 induces antigen specific antibody response

**Figure 4:**
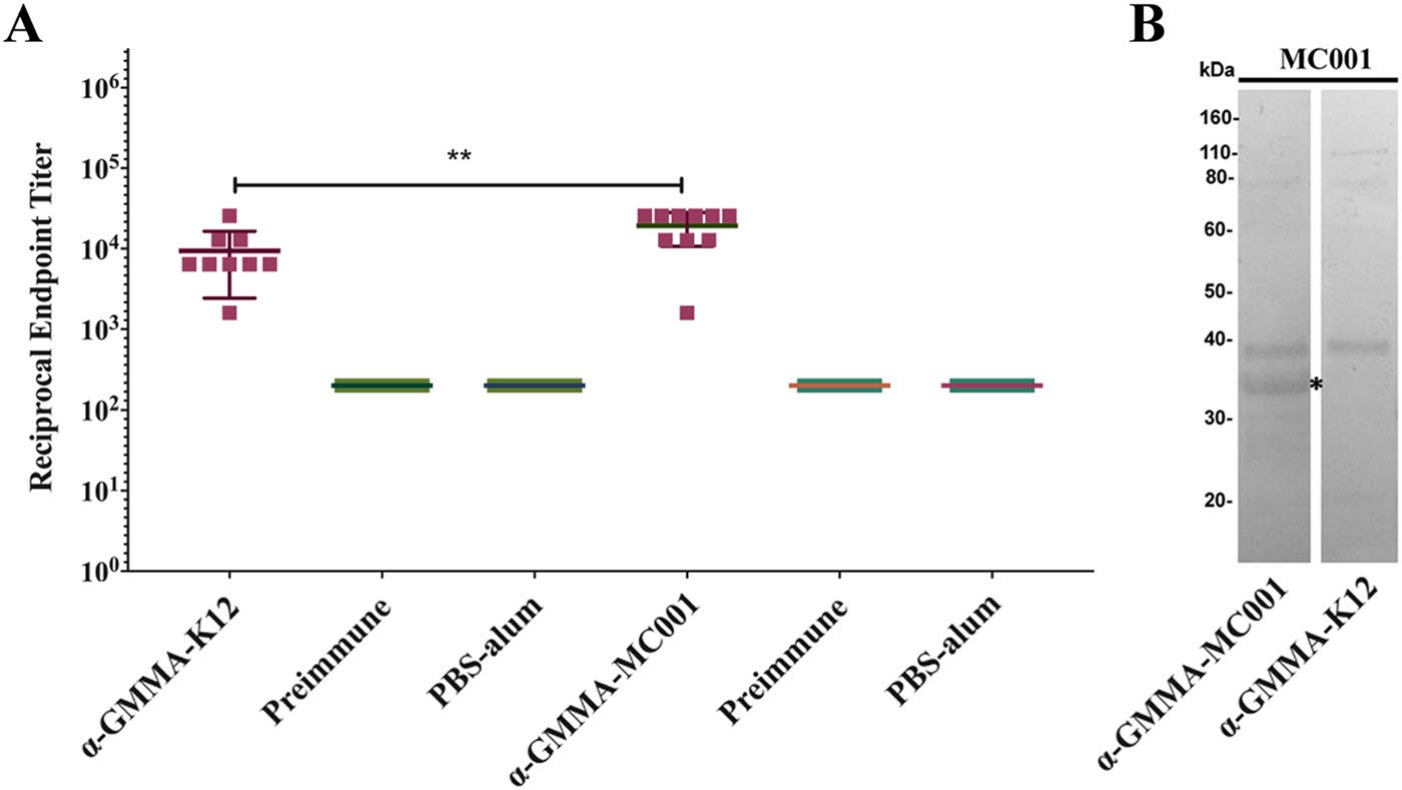
Detection of specific antibodies raised against GMMA overexpressing the MC001 vaccine candidate. (**A)** Specific antibody (IgG) measurement of sera obtained by immunization of GMMA-K12 and GMMA overexpressing MC001 was assessed by an ELISA assay using microtiter plates coated with the MC001 recombinant protein. The plots represent individual average. Data are expressed as means ± the Standard Deviation of values from 9 mice for GMMA-MC001 and 10 mice for GMMA-K12 group and asterisks (**) indicate statistically significant differences (*P* value < 0.05) (*P* value = 0.0076) calculated by Mann-Whitney U test. Student t tests, indicates a significant difference in reciprocal endpoint titers between mice vaccinated with GMMA-K12 (control group) and GMMA overexpressing the vaccine candidate. Pre-immune sera and mice immunized with PBS-alum were used as negative controls. The endpoint titer of a sample is defined as the reciprocal of the highest dilution that has a reading above the cutoff. **(B)** Western blot assay using MC001 recombinant protein as target. Sera raised against GMMA overexpressing the vaccine candidates and GMMA-K12 (negative control) were used for detection. The asterisk (*) indicates the expected molecular size.

To assess the immune response induced by GMMA immunization *per se* and the specific contribution of MC001 vaccine candidate overexpressed in GMMA, mice serum antibody levels were determined by ELISA. Serum samples were collected prior to the first immunization (preimmune sera) and two weeks after the third immunization, before challenging mice with the EHEC O157:H7 86–24 stain. Microtiter plates were coated with purified preparations of GMMA-K12 or with GMMA expressing the MC001 vaccine candidate. Higher total IgG levels were measured in the immunized group *versus* the preimmune sera or PBS-alum immunized mice. No-significant differences in total IgG were found among the mice immunized with empty GMMA-K12 in comparison with GMMA expressing MC001 (Fig. S2), suggesting that the presence of the antigen does not interfere with the immunogenicity of the GMMA. To test whether there was an induction of a specific immune response attributable to the antigen expressed in GMMA, antibodies against MC001 were measured by the ELISA assay using the recombinant MC001 as coating antigen. A significant increase in antibody response was found in sera of mice immunized with GMMA-MC001 (*P* = 0.0076) in comparison to GMMA-K12 (Fig. 4A). Furthermore, the sera raised against GMMA-MC001 were able to specifically detect the MC001 recombinant protein by western blot analysis (Fig. 4B).

### Immunization with MC001-GMMA reduces EHEC intestinal bacterial colonization in mice

**Figure 5:**
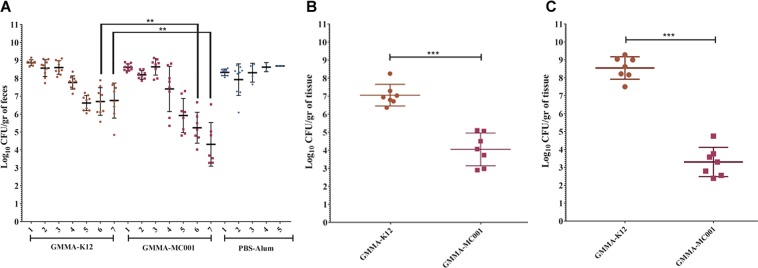
EHEC intestinal colonization model using mice immunized with GMMA overexpressing vaccine candidates. (**A**) Graphic representation of 7-days bacterial counts in feces of immunized mice with GMMA vaccine overexpressing the candidate (GMMA-MC001) or with empty GMMA-K12 or PBS-alum via intraperitoneal at day 1, 21 and 39. Mice were challenged using EHEC O157:H7 86–24 strain via gavage. Fecal samples were collected daily, performing bacterial counts represented as CFU per gram of feces (CFU/gr). The plots represent individual CFU counts (dots). Horizontal bars represent geometric mean ± the standard deviation (SD) of values from the mice in each group. Asterisks indicate statistically significant differences (*P* value < 0.05) between GMMA-MC001 and GMMA-K12 groups comparison (**P value = 0.0033 at day 6 and **P value = 0.0037 at day 7) calculated by Mann-Whitney U test. (**B**) Graphic representation of bacterial counts in colon tissue (CFU/gr) of immunized mice (GMMA-MC001 compared to GMMA-K12, ***P value = 0.0006). (**C**) Graphic representation of bacterial counts in caecum tissue (CFU/gr) of immunized mice (GMMA-MC001 compared to GMMA-K12, ***P value = 0.0006).

The ability of the MC001 candidate to induce a protective immune response was evaluated in the mouse EHEC intestinal colonization model. Groups of ten BALB/c mice were immunized intraperitoneally at day 1, 21 and 39 with GMMA overexpressing the candidate or with native GMMA-K12 formulated with alum-hydroxide or with buffer (PBS-Alum) alone used as negative control. At day 50, mice were infected with the EHEC O157:H7 86–24 strain (5 × 10^9^ CFU), *via* gavage. Fecal samples were collected daily to perform bacterial counts. GMMA-MC001 immunized mice showed a ≈2.7-log reduction in bacterial number at day 5 compared to mice immunized with PBS-alum, while a ≈0.6-log, ≈1.3-log and ≈2.4-log reduction at day 5, 6 and 7 respectively was observed in comparison to mice immunized with GMMA-K12. Significant differences (*P* value < 0.05) were only observed at day 6 and 7 (**P value = 0.0033 and **P value = 0.0037 respectively) in the GMMA immunized mice (Fig. 5A). For ethical reasons, at day 5 most of the PBS-alum immunized mice were euthanized due to weight loss (>15% of initial body weight). In addition, at day 7 post infection, colon and caecum tissues were collected from all mice and adherent bacteria counted. The number of bacteria in colon and cecum tissues was significantly reduced (≈3-log and ≈5-log, ***P value = 0.0003) in mice immunized with the GMMA-MC001 in comparison to GMMA-K12 (Fig. 5B,C).

### MC001 has homology with the lipid A deacylase (LpxR), and its gene is highly conserved among EHEC

**Figure 6:**
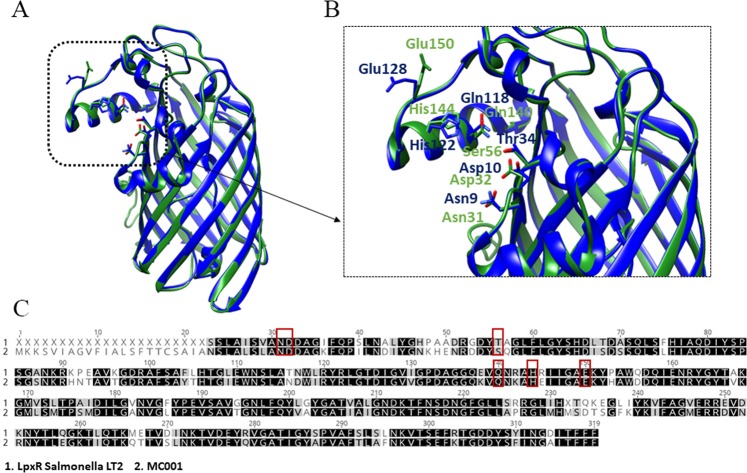
MC001 comparative protein modeling. (**A**) Structural model of MC001 obtained by SWISS-MODEL. The green ribbon represents the 12-stranded β-barrel structural model of MC001 based on LpxR from *Salmonella* serovar Typhumurim three-dimensional structure (PDB: 3FID), represented by the blue ribbon. (**B**) Enlarged view of the catalytic site region showing conserved residues for Ca^+2^ binding and LpxR catalytic activity: Asn (9/31), Asp (10/32), Thr/Ser (34/56), Gln (118/140), His (122/144) and Glu (128/150). (**C**) Sequence alignment of MC001 and LpxR. The proteins display an amino acid sequence identity of 74% and a similarity of 93%. Conserved residues are highlighted in black, conservatively substituted ones in grey. The residues of the active site are marked by red squares.

To obtain further insight into the structural features of the MC001 candidate, an *in-silico* analysis was performed. In order to find proteins with known structure and significant sequence similarity with MC001, its protein sequence was used to run a PSI-BLAST search against the Protein Data Bank (PDB)^32^. This search retrieved the sequence of the lipid A deacylase (LpxR) from *Salmonella* Typhimurium with high confidence. Furthermore, a PSI-BLAST search of the non-redundant protein sequences database also revealed high sequence similarity with LpxR from *Vibrio cholerae, Yersinia enterocolitica* and *Helicobacter pylori*. Structural MC001 models using the LpxR structure as a template (PDB code: 3FID)^33^ were built by I-TASSER, MEMOIR and SWISS-MODEL software. All three generated models were similar, showing a pairwise Cα root mean square deviation in all cases lower than 0.5 Å (Fig. 6A). The MC001 structural model is composed of a 12-stranded β-barrel, in which the β-strands are arranged in an antiparallel fashion similarly to the common fold of porins and other cell membrane proteins. The high structural similarity between MC001 and *Salmonella* Typhimurium LpxR was confirmed by the presence of six conserved residues in the active site (Asn (9/31), Asp (10/32), Thr/Ser (34/56), His (122/144), Gln (118/140) and Glu (128/150). These residues are important for Ca^2+^ binding, which is essential for LpxR catalytic activity (Fig. 6B). Sequence comparison revealed that MC001 is the same length of the LpxR *Salmonella* ortholog (319 amino acids) and shares 74% sequence identity and 94% sequence similarity (with 93% of sequence coverage) (Fig. 6C). Furthermore, to extend the MC001 gene prevalence and sequence variability analysis a dataset of 224 EHEC genomes, frequently associated with human infections and outbreaks, including O157:H7 and the big six non-O157 serotypes (O26:H11, O45:H2, O103:H2, O111:H8, O121:H19, O145:H28), was queried in a BLAST search. The results showed that MC001-encoding gene was present in 97.3% (218 of 224) of the genomes with 99.7–100% sequence identity (query coverage ≥ 85%) (Table S5). The *lpxR* gene thus appears to be prevalent and highly conserved among the main serotypes associated with EHEC infection.

## Discussion

The great diversity of *E. coli* genomes has made the identification of broadly conserved antigens unique to the intestinal pathovar difficult. In this study, the reverse vaccinology approach was applied to identify new vaccine candidates in intestinal pathogenic *E. coli*, focusing on EHEC. In recent years, this methodology has been exploited as an *in silico* tool to discover new protein antigens for several pathogens with success^11–15^.

An important characteristic for a vaccine candidate is its ability to confer broad-spectrum protection against most circulating strains. Recently, a novel antigen (YncE) has been proposed as universal vaccine candidate against all pathogenic *E. coli* strains^34^. By contrast, antigens such as SslE, a conserved and protective antigen identified in ExPEC, was found to be absent in EHEC and other intestinal pathotypes^15^. Similarly, other proposed vaccine candidates, such as LEE and Stx, are not present in all InPEC strains, nor even in all non-O157 EHEC strains, therefore limiting the vaccine use of these important virulence factors^10^.

In this study, we focused on chromosomally-encoded proteins predicted to be secreted, surface-exposed and outer membrane-associated, as these are more accessible to antibodies and therefore representing ideal vaccine candidates^35,36^. In addition, we evaluated whether these putative candidates were effectively expressed *in vitro* and *in vivo* during infection, by measuring their gene expression level. By combining genomic analysis with transcriptional and molecular epidemiology data, it was possible to identify 24 new vaccine candidates present and conserved in more than one intestinal pathotype that differed from the antigens previously described^11^.

GMMA expression of MC001 allowed delivery of the antigen in a membrane-associated form, increasing the ability to induce a functional immune response. Expression in GMMA has already been successfully applied to many heterologous membrane associated antigens^24–26,30^. Since native proteins are present on the membrane surface, GMMA can potentially act as self-adjuvants, helping to elicit immune responses^37^. However, in native conditions, blebs are produced in small quantities and, as a consequence, *E. coli* strains need to be genetically modified to increase the amount of outer membrane produced. For example, deletion of the *tolR* gene results in an *E. coli* strain with an increased level of vesicles production^23,27,30^. Recently, reduced-toxicity outer membrane vesicles from a virulent STEC (Shiga toxin *Escherichia coli*; O157:H7 serotype), obtained by detergent extraction and chemical inactivation, have shown a protective effect against STEC challenge in a murine model and are immunogenic in calves^38^.

In this study, we tested the ability to prevent or reduce bacterial infection of the selected candidate when expressed in GMMA^11,39,40^. Although mice did not develop the symptoms associated with the diarrheal disease observed in humans, these murine models of *E. coli* O157:H7 infection, based on streptomycin-treated BALB/c mice, have been promising for EHEC colonization and candidate vaccine testing^39,40^. Immunization with GMMA MC001 induced antibodies able to recognize the MC001 antigen and reduce bacterial colonization in the feces, colon and cecum. These data suggest that, for a functional antigen delivery into the GMMA, the heterologous antigen needs to be expressed and properly presented to the immune system.

Furthermore, a bioinformatic analysis revealed that MC001 is homologous to the *Salmonella* Typhimurium lipid A deacylase (LpxR) and shares similarity also with LpxR from *Vibrio cholerae, Yersinia enterocolitica* and *Helicobacter pylori*. It has been reported that LpxR can play an important role in pathogenesis by removing the 3′-acyloxyacyl group from lipid A (the hydrophobic anchor of lipopolysaccharide, LPS). This modification increases the ability of *Salmonella* Typhimurium to evade the innate immune response and promotes its survival within macrophages^41,42^. Interestingly, the role of LpxR in innate immune response evasion has recently been elucidated. Ogawa and colleagues have shown that increased inflammatory and phagocytic responses were induced by the *lpxR* mutant EHEC O157:H7 Sakai strain with reduced lipid A deacylation^43^. These effects were attributed to augmented NF-κB and phosphorylated p38 mitogen protein kinase (MPK) signaling, both *via* a TLR4 response. In contrast, LpxR-positive strains able to modify the lipid A were capable of attenuating these pathways. As shown in Fig. S1 (and by Ogawa and colleagues^43^) the LpxR gene is also present in other LEE positive pathotypes such as EPEC in which expression of LEE genes and non-LEE effector encoding genes is activated through the effect of phage-encoded (Pch) and LEE-encoded regulators (Ler). However, adhesion mediated by T3SS was not affected by mutation of *lpxR* indicating that this gene indirectly contributes to colonization by the reduction of the inflammatory response, and this is important for attachment of EPEC/EHEC to the mucosal host cells^43^. In this regard, raising a specific antibody response toward LpxR could potentially bypass LPS modification and subsequent immune evasion. However, further studies will be required to better understand the mechanism of action of the LpxR immune response in EHEC colonization and pathogenesis. Interestingly, the gene variability and distribution analysis in a large genomes dataset showed that MC001 was highly prevalent and conserved among EHEC belonging to serotypes O157:H7, as well as in the big six non-O157 serotypes (O26:H11, O45:H2, O103:H2, O111:H8, O121:H19, O145:H28), which are the strains most frequently associated with human disease and outbreaks globally^10^. Therefore, given the high genetic variability among and within these pathotypes, the identification and inclusion of this conserved candidate in a vaccine might offer protection against major intestinal pathogenic strains associated with human diseases. In conclusion, the reverse vaccinology approach applied on the EHEC O157:H7 genome combined with the GMMA antigen delivery system provides a cost-effective strategy for identifying a novel promising vaccine candidate.

## Material and Methods

### Bacterial strains and culture conditions

All bacterial strains were routinely grown at 37 °C in Lysogeny Broth (LB) media and with supplemented with antibiotics if required. The *E. coli* Mach1-T1R (Thermofisher) strain was used for cloning while BL21-λDE3 (NEB) strain was used to express and purify the antigens as recombinant proteins. EDL933 is the prototype strain of the EHEC O157:H7 pathotype used in this study for antigen identification by the reverse vaccinology approach. The strain EHEC O157:H7 86–24 was used for animal challenge experiments.

### Vaccine candidate selection by reverse vaccinology

*In silico* vaccine candidate identification was performed as follows. The 5675 CDSs (coding sequences) of the annotated EHEC O157:H7 EDL933 strain genome (GeneBank sequence CP008957.1) were analyzed by PSORT software^44^ to predict the subcellular localization. HTMM was used for prediction of transmembrane regions in putative proteins^45^. RNA-Seq mapping and RPKM (Reads Per Kilobase Million) calculation (cutoff > 10) was performed using Geneious R9 software (http://www.geneious.com^46^. Distribution and sequence variability analysis in *E. coli* genomes was performed by BLASTP^47^ using a cutoff of ≥90% of query coverage and a ≥80% of sequence protein identity. Only antigens present in more than 5 intestinal pathogenic *E. coli* strains were selected.

### Cloning and recombinant protein production of vaccine candidates

All candidates were cloned and expressed as His-tagged fusion proteins without their predicted signal sequence. Prediction of the signal peptide was performed by Signal P^48^. All fragments were amplified by PCR using primers listed in Table S4 and using genomic DNA of *E. coli* EHEC O157:H7 EDL933 strain as the template. The PCR amplicons were cloned into pET-15b (Novagen, EMD Millipore) with a His-tag in the carboxyl-terminus by the polymerase incomplete primer extension (PIPE) method^49^ or the NEBuilder HiFi DNA Assembly Master Mix (NEB). Plasmids were transformed in BL21-λDE3 (NEB). Briefly, *E. coli* BL21-λDE3 harboring pET-15b constructs were grown in EnPresso medium (Sigma Aldrich) following the manufacturer suggestions using 0.01 M IPTG (Sigma Aldrich) for induction. After centrifugation, cell pellets were lysed by sonication. The suspension obtained was centrifuged and the supernatant passed through a Ni^2+^-NTA agarose column (Qiagen). Proteins were eluted using imidazole-concentration gradient buffers. In the case of insoluble proteins, the imidazole buffers for purification contained urea 6 M urea. Protein concentration was measured using the Pierce BCA Protein Assay Kit (Thermofisher).

### Construction of TolR mutant

The *tolR* mutant in *E. coli* K12 MC4100 strain was constructed by allelic maker exchange using the Lambda red system^50^. The *tolR* gene was interrupted with a chloramphenicol resistance cassette (*cat*). Briefly, the *cat* cassette was amplified using forward and reverse primers with ≈70-nucleotides tails homologous to the flanking regions of *tolR* (Table S4). The PCR product was purified and used to transform *E. coli* K12 recipient cells (carrying the plasmid expressing the recombinase E, pKD46) as previously described^50^. The deletion of the *tolR* gene was confirmed by PCR using primers flanking *tolR*.

### NOMV and GMMA production

For native OMV (NOMV) isolation, the *E. coli* K12 MC4100 WT strain was grown at 37 °C in liquid LB medium. For GMMA production, *E. coli* K12 ∆*tolR*::*cat* was grown at 37 °C in liquid LB medium containing chloramphenicol (20 µg/ml) as previously described^26,51^. Briefly, 75 ml of media were inoculated with *E. coli* K12 wild-type (wt) or ∆*tolR*::*cat* and grown at 37 °C, 150 rpm overnight (≈16 h). To recover the supernatants cultures were centrifuged for 30 min at 8, 000 × *g* and 0.22 µm filtered. These media were ultracentrifuged using propylene ultracentrifuge tubes (Beckman Coulter) at 105,000 × *g* for 2 hours at 4 °C. Pellets were washed once with phosphate-buffered saline (PBS) and centrifuged once more. Finally, pellets were resuspended in 2 ml of PBS followed by 0.22-µm filtration and vesicles were stored at 4 °C. To determine the total protein content present in these preparations, quantification was performed by DC protein assay (Bio-Rad) based on the Lowry assay^52^.

### Negative-staining transmission electron microscopy

One drop of 10 µL of GMMA or NOMV suspension was placed on copper formvar/carbon-coated grids and adsorbed for 2 min. Grids were then washed with few drops of distilled water and blotted with Whatman filter paper. For negative staining, grids were treated with Uranyless EM stain (Delta Microscopy, France) for 1 min, air-dried and viewed with a Hitachi H-7650 transmission electron microscope at 80 kV. Electron micrographs were recorded at a nominal magnification of 120, 000x.

### Over-expression of antigens in GMMA

To overexpress MC001 candidate in GMMA, the corresponding coding sequence, including its native signal peptide, was cloned into frame into pBAD-A (Thermofisher) using the NEBuilder HiFi DNA Assembly Master Mix (NEB). Further, a DNA sequence encoding a FLAG-tag (DYKDDDDK) was introduced between the sequence encoding the native signal peptide and the rest of the protein. The generated constructs (pBAD-MC001F), was transformed into the *E. coli* K12 ∆*tolR*::*cat* mutant and induced with arabinose (0.01% final concentration). The purified GMMA were named GMMA-MC001. GMMA not expressing antigen and obtained by transforming the empty pBAD-A plasmid were named GMMA-K12.

### Mice immunization and colonization model

For the challenge experiment, five weeks-old BALB/c mice (Janvier) (10 mice per group) were immunized with GMMA-MC001, or GMMA-K12 adjuvanted with 2 mg/ml alum hydroxide (Alhydrogel 2%, Invivogen) or with PBS-alum. Animals were immunized by intraperitoneal injections (i.p.) with 10 µg of GMMA plus adjuvant at day 1 and with 5 µg of GMMA plus adjuvant at day 21 and day 35. Blood was collected from all the mice prior immunization and two weeks after the third dose. The challenge experiment was performed 2 weeks after the last immunization using the EHEC O157:H7 strain 86–24. Mice were treated with streptomycin 24-hours prior infection. Animals additionally received cimetidine 2 hours before infection *via* i.p. Animals were infected with 5 × 10^9^ CFU via gavage. Animal monitoring was performed daily monitoring weight, signs and symptoms. According to ethical rules, mice displaying signals of illness and losing more than 15% of the total weight were euthanized, collecting the caecum and colon. Fecal pellets were collected every day from day 1 to day 7 post-infection. At day 7 the remaining mice were sacrificed, and organs were collected. This animal model was adapted from models previously reported^11,39,40^. All animal experiments were reviewed and approved by the Auvergne Ethical Committee for Animal Experimentation C2EA (Agreement N°6065-2016071216144325V2). For specific polyclonal antibodies generation purified recombinant proteins, adjuvanted with 2 mg/ml alum hydroxide, were used for subcutaneous immunization of CD-1 outbred mice (Charles River). These immunization experiments were performed at the Novartis Vaccines Animal Facility in Siena, Italy, (now acquired by the GSK group) in compliance with the relevant guidelines of Italy (Italian Legislative Decree n. 116/1992) and the institutional policies of Novartis (now acquired by the GSK group). The animal protocol was approved by the Animal Welfare Body of Novartis Vaccines, Siena, Italy, (now acquired by the GSK group) and by the Italian Ministry of Health (Approval number AWB2012-03).

### Enzyme-linked immunosorbent assay (ELISA)

Ninety-six well Maxisorp plates (Nunc, Thermo Fisher Scientific) were coated with 1 µg/ml of GMMA preparations expressing antigen or 1 µg/ml of recombinant protein in PBS overnight (O/N) at 4 °C. The next day, plates were washed 3 times with T-PBS (0.05% Tween-20 in PBS, pH 7.4) and blocked with 100 µl 2% BSA (Sigma Aldrich) for 1 hour at 37 °C. Each incubation step was followed by triple T-PBS wash. Serum samples were initially diluted 1:200 in 2% BSA in T-PBS, transferred to coated-blocked plates and serially 2-fold diluted followed by a 2-hours incubation at 37 °C. Then 100 µl/well of alkaline phosphatase-conjugated goat anti-mouse IgG (H + L) (Southern Biotech) 1:2,000 diluted were added and incubated for 2 hours at 37 °C. Bound alkaline phosphatase was visualized by adding SIGMAFAST p-Nitrophenyl phosphate (Sigma Aldrich). After 30 minutes at room temperature, plates were analyzed at 405 nm in a microplate spectrophotometer. The endpoint titer of a sample is defined as the reciprocal of the highest dilution that has a reading above the cut-off using the formula described by Frey and collaborators^53^.

### Western blotting

Western blots were carried out on whole cell extracts (wce), recombinant proteins or GMMA preparations. SDS-PAGE was performed in MES buffer (Thermofisher) and proteins were transferred to iBlot 2 nitrocellulose stacks (iBlot system, Thermofisher). To visualize transferred proteins, the membranes were stained with Ponceau red. Then, membranes were blocked with 10% (w/v) blotting-grade blocker (Bio-Rad) in T-PBS. The membranes were later incubated with the respective mouse polyclonal antisera in a 1:1000 dilution in T-PBS-3% blocker 1 h at room temperature. Membranes were washed three times with T-PBS and then incubated with goat anti-mouse horseradish peroxidase-conjugated IgG (Dako antibodies) diluted (1:2000) in T-PBS-3% blocker. Colorimetric staining was performed using Opti-4CN Substrate Kit (Bio-Rad) following manufacturer instructions. To detect the FLAG-tag, the monoclonal ANTI-FLAG M2 secondary antibody was used (Sigma Aldrich).

### Comparative structural modelling

Structural models of MC001 have been obtained by employing three different approaches: the threading/*ab initio* modelling method implemented in the I-TASSER pipeline^54^, the membrane proteins-specific approach of MEMOIR^55^ and the homology modelling method of SWISS-MODEL^56^. The search for suitable modelling templates has been carried out with PSI-BLAST (Position-Specific Iterated BLAST)^47^ sequence similarity search against the Protein Data Bank using the amino acid sequence of MC001 as a bait. While MEMOIR does not provide a proper quality assessment of the models, in the case of I-TASSER and SWISS-MODEL, the quality of the final models has been assessed through the parameters C-score and QMEAN4^57^, respectively. The C-score is a confidence score calculated based on the reliability of threading template alignments and the convergence parameters of the structure assembly simulations. C-score values typically range between −5 and 2, higher values characterizing high confidence models and vice-versa. The QMEAN4 score is a linear combination of four statistical potential terms and is typically in the range 0–1, with higher values characterizing better quality models. MC001 models are characterized by a C-score of −5 and a QMEAN4 score of 0.74.

### Statistical analysis

Statistical analysis was performed by using GraphPad Prism 7 software. Mann-Whitney (unpaired and non-parametric) and Student t tests with threshold of *P* < 0.05 was used to analyze the data of the bacterial counts from the mouse colonization model and for the IgG antibody response.

### Ethical approval

Animal experiments at INRA were reviewed and approved by the Auvergne Committee for Animal Experimentation C2EA (Agreement N°6065-2016071216144325V2). Immunization experiments at the Novartis Vaccines Animal Facility in Siena, Italy, (now acquired by the GSK group) were performed in compliance with the relevant guidelines of Italy (Italian Legislative Decree n. 116/1992) and the institutional policies of Novartis (now acquired by the GSK group). The animal protocol was approved by the Animal Welfare Body of Novartis Vaccines, Siena, Italy, (now acquired by the GSK group) and by the Italian Ministry of Health (Approval number AWB2012-03).

## Supplementary information

Supplementary_information_Rojas-Lopez_et_al

Table S1

Table S2

Table S3

Table S4

Table S5

## Data Availability

The datasets generated during and/or analyzed during the current study are available from the corresponding author on reasonable request.
