# Supplementary material for: Identification of lipid A deacylase as a novel, highly conserved and protective antigen against enterohemorrhagic Escherichia coli
Source: Sci Rep. 2019 Nov 19;9:17014. doi: 10.1038/s41598-019-53197-z (PMC6863877; doi:10.1038/s41598-019-53197-z)
Supplement: Supplementary file 1 — Supplementary_information_Rojas-Lopez_et_al [file 41598_2019_53197_MOESM1_ESM.pdf]

Identification of lipid A deacylase as a novel, highly conserved and protective antigen against enterohemorrhagic *Escherichia coli*

Maricarmen Rojas-Lopez[1,2,3], Manuele Martinelli[1], Valentina Brandi[3], Grégory Jubelin[2], Fabio Polticelli[3,4], Marco Soriani[1,5], Mariagrazia Pizza[1], Mickaël Desvaux[2], Roberto Rosini[1]*.

[1] GSK, Via Fiorentina 1, 53100, Siena, Italy.

[2] Université Clermont Auvergne, INRA, UMR454 MEDiS, F-63000 Clermont-Ferrand, France.

[3] Department of Medicine, Division of Infectious Diseases, Massachusetts General Hospital; Department of Microbiology and Immunobiology, Harvard Medical School, Boston, Massachusetts, USA.

[3] Roma Tre University, Department of Sciences, Viale G. Marconi 446, 00146 Rome, Italy.

[4] National Institute of Nuclear Physics, Roma Tre Section, Via della Vasca Navale 84, 00146 Rome, Italy.

[5]ReiThera Srl, Via di Castel Romano 100, 00128 Roma, Italy

*Corresponding author: Roberto Rosini, GSK, Via Fiorentina 1, 53100, Siena, Italy Tel.: +39 05 77 25 38 88, Fax: +39 05 77 25 38 88, e-mail: roberto.x.rosini@gsk.com

Vaccine candidate name	Intestinal *Escherichia coli*																	Extraintestinal *Escherichia coli*													
	EHEC							EPEC		ETEC			AIEC			EAEC		NMEC			UPEC								AREC	APEC	ABU
	EHEC O157:H7 TW14359	Sakai	EC4115	EDL933	O26:H11 Str. 11368	O103:H2 Str. 12009	O111:H- Str 11128	EPEC O55:H7 CB9615	O127:H6 Str E2348/69	ETEC H10407	E24377A	UMNK88	AIEC LF82	O83:H1 NR G857C	UM146	EAEC 042	55989	NMEC O7:K1 CE10	S88	IH E3034	UPEC UMN026	CLONE Di14	CLONE Di2	CFT073	IAI39	536	NA114	UTI89	AREC SMS-3-5	APEC01	ABU 83972
MC001	■	■	■	■	■	■	■	■	■																						
MC002	■	■	■	■	■	■	■	■																							
MC003	■	■	■	■				■								■		■			■				■				■		
MC004	■	■	■	■		■	■																								
MC005		■		■	■	■	■																								
MC006	■	■	■	■				■																							
MC007	■	■	■	■				■																							
MC008	■	■	■	■				■																							
MC009	■	■	■	■				■																							
MC010	■	■	■	■				■																							
MC011	■	■	■	■				■																							
MC012	■	■	■	■																											
MC013	■	■	■	■				■								■		■			■				■				■		
MC014	■	■	■	■																											
MC015	■	■	■	■				■																							
MC016	■	■	■	■				■																							
MC017	■	■	■	■	■		■						■																		
MC018	■	■	■			■		■		■																					
MC019	■	■	■	■																											
MC020	■	■	■	■				■		■		■				■					■								■		
MC021	■	■	■	■				■											■										■		
MC022	■	■	■	■				■								■		■			■				■				■		
MC023	■	■	■	■		■	■	■									■														
MC024	■	■	■	■				■																							

Figure S1. The selected candidates are mainly present and conserved among EHEC strains. Gene variability and distribution analysis (by BLASTP) shows the presence/absence of the antigens in a panel of 31 *E. coli* InPEC and ExPEC complete genomes. Hits are represented by Black cells (sequence identity ≥ 80%, query coverage ≥ 90%) white cells represent gene absence or presence with a sequence identity <80% and query coverage < 90%. EHEC: enterohemorrhagic *E. coli*; EPEC: enteropathogenic *E. coli*; ETEC: enterotoxigenic *E. coli*; EIEC: enteroinvasive *E. coli*; EAEC: enteroaggregative *E. coli* (EAEC); NMEC: neonatal meningitis *E. coli;* UPEC: uropathogenic *E. coli*; AREC: antibiotic resistant *E. coli;* APEC avian pathogenic *E. coli*; ABU: Asymptomatic *E. coli*. ***E. coli* O111:H- str. 11128** (EHEC,

NC_013364.1); ***E. coli*** **O157:H7 str. TW14359** (EHEC, NC_013008.1); ***E. coli*** **O157:H7 str. Sakai** (EHEC, NC_002695.1); ***E. coli*** **O157:H7 str. EC4115** (EHEC, NC_011353.1); ***E. coli*** **O157:H7 EDL933** (EHEC, NC_002655.2); ***E. coli*** **O103:H2 str. 12009** (EHEC, NC_013353.1); ***E. coli*** **O26:H11 str. 11368** (EHEC, AP010953.1); ***E. coli*** **O127:H6 str. E2348/69** (EPEC, NC_011601.1); ***E. coli*** **O55:H7 str. CB9615** (EPEC, NC_013941.1), ***E. coli*** **UMNK88** (ETEC, CP002729.1); ***E. coli*** **strain H10407** (ETEC, NC_017633.1); ***E. coli*** **E24377A** (ETEC, NC_009801.1); ***E. coli*** **UM146** (AIEC, CP002167.1); ***E. coli*** **O83:H1 str. NRG 857C** (AIEC, CP001855.1); ***E. coli*** **LF82** (AIEC, CU651637.1); ***E. coli*** **55989** (EAEC, NC_011748.1); ***E. coli*** **042** (EAEC, FN554766.1); ***E. coli*** **S88** (NMEC, CU928161.2); ***E. coli*** **O7:K1 str. CE10** (NMEC, CP003034.1); ***E. coli*** **IHE3034** (NMEC, CP001969**); *E. coli*** **CFT073** (UPEC, AE014075), ***E. coli*** **UTI89** (UPEC, NC_007946.1), ***E. coli*** **UMN026** (UPEC, NC_011751.1); ***E. coli*** **str. 'clone D i14'** (UPEC, CP002212.1); ***E. coli*** **str. 'clone D i2'** (UPEC, CP002211.1); ***E. coli*** **NA114** (UPEC, CP002797.2); ***E. coli*** **IAI39** (UPEC, NC_011750.1), ***E. coli*** **536** (UPEC, NC_008253.1); ***E. coli*** **SMS-3-5** (AREC, NC_010498.1), ***E. coli*** **APEC O1** (APEC, NC_008563); ***E. coli*** **ABU 83972** (ABU, CP001671.1).

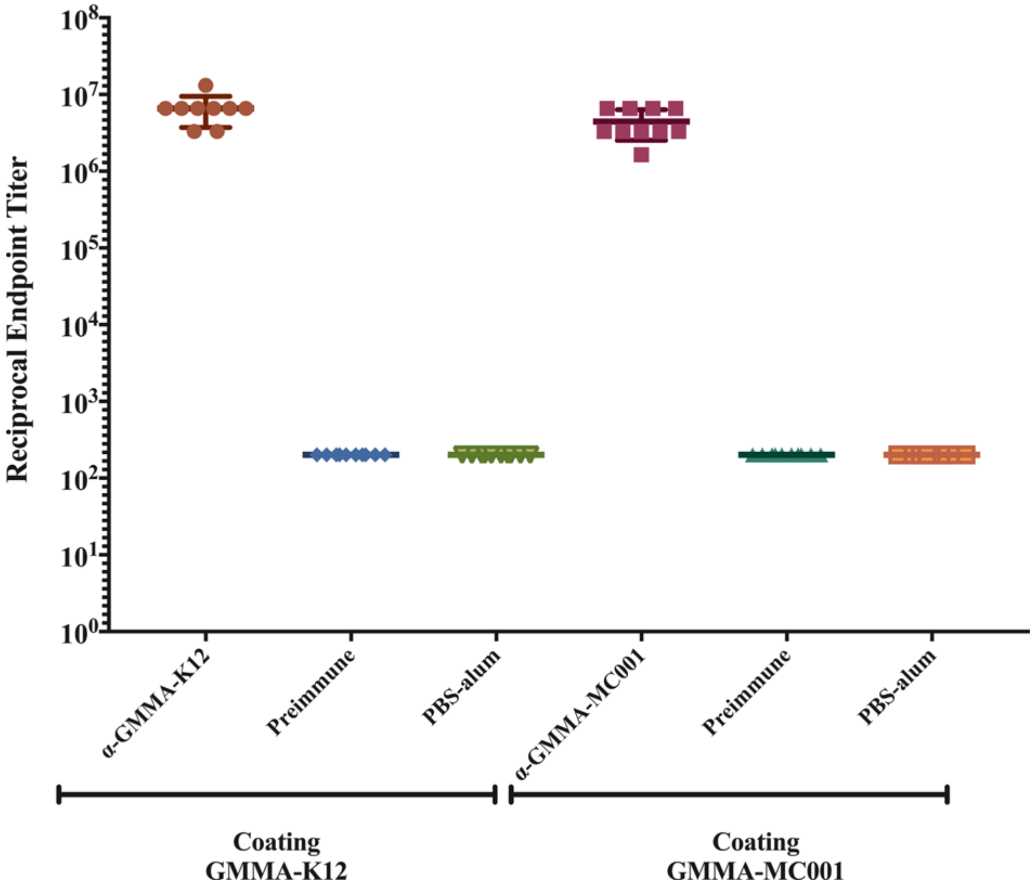

Figure S2. Total IgG response after immunization with GMMA-K12 and GMMA-MC001. (A) IgG antibody relative titers of GMMA overexpressing MC001 were measured by ELISA assay. Microtiter plates were coated with purified preparations of each GMMA-K12 and GMMA carrying the vaccine candidates. Sera raised against the two groups were collected from vaccinated animals two weeks post the third immunization. Pre-immune sera and mice immunized with PBS-alum were used as negative controls. The plots represent individual average. Data are expressed as means ± the SD of values from ten mice in each group. The endpoint titer of a sample is defined as the reciprocal of the highest dilution that has a reading above the cutoff.

Original gels and blots images

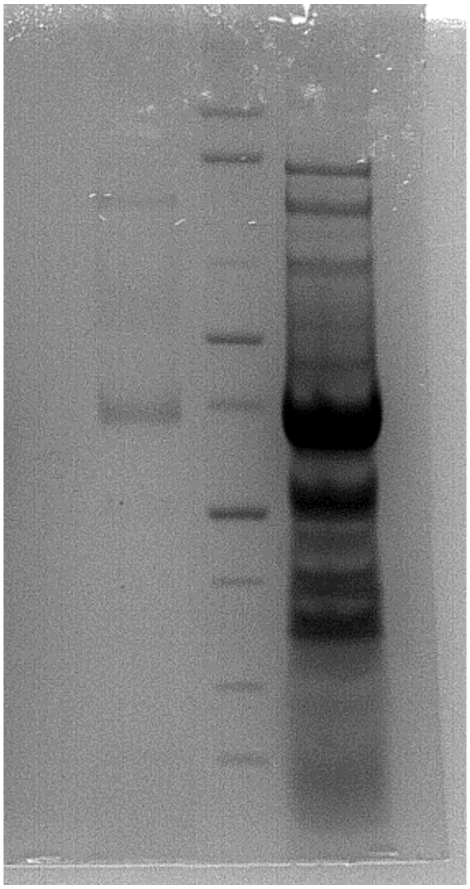

Figure 2. *E. coli* K12 engineering to generate Generalized Modules for Membrane Antigens (GMMA). Original gel image. SDS Page (4-12% bis-tris polyacrylamide) of membrane vesicles (NONV and GMMA) each purified from 75 ml of culture supernatants. Total protein content was quantified and 50 ug of GMMA obtained from K12 Δ*tolR::cat* sample was loaded into the SDS-PAGE gel. An equivalent volumetric amount of NOMV from K12 WT obtained from 75 mL of supernatant was loaded. The *tolR* mutant showed an extensive protein profile in the supernatant compared to wild type.

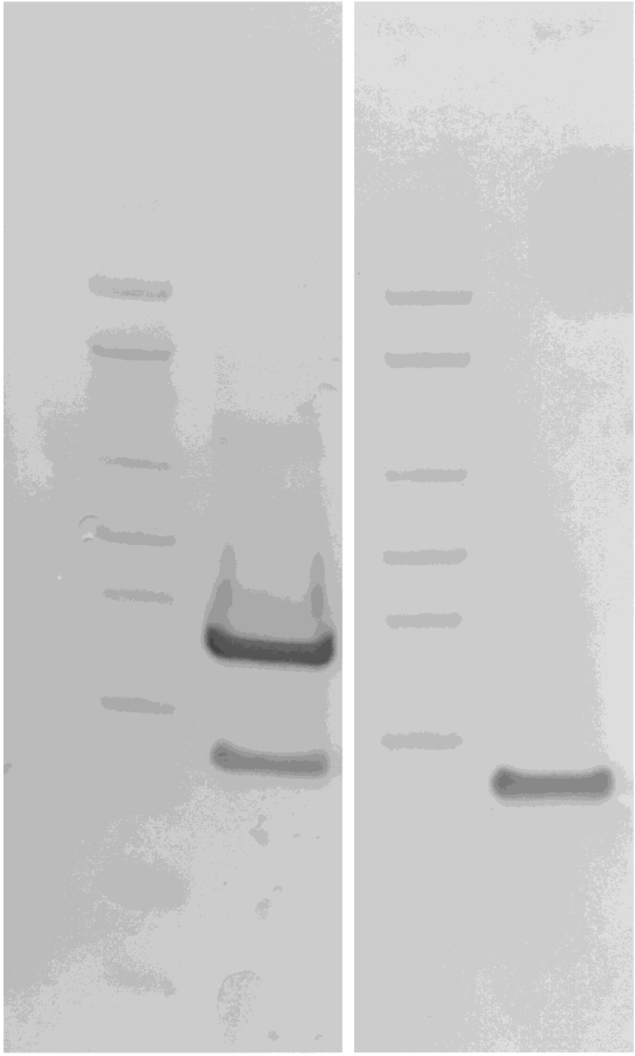

Figure 3. Antigen delivery into GMMA. Original western blot picture. Western blot of GMMA preparation expressing MC001 candidate purified from the K12 *tolR::cat* mutant using an anti-FLAG antibody.

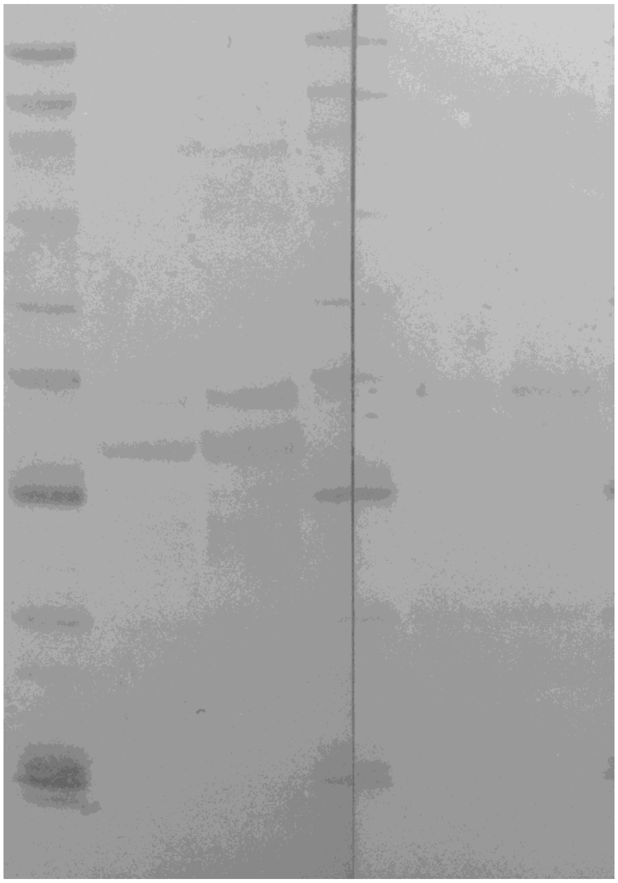

Figure 4. Detection of specific antibodies raised against GMMA overexpressing MC001 vaccine candidate. Original western blot picture. Western blot assay using MC001 recombinant protein as target. Sera raised against GMMA overexpressing the vaccine candidates and GMMA-K12 (negative control) were used for detection.
